# A Remarkable Case of Acute Stroke Unveiling Congenitally Corrected Transposition of Great Arteries

**DOI:** 10.7759/cureus.54889

**Published:** 2024-02-25

**Authors:** Sweta Sahu, Varnika Gupta, Mahendra Kumar, Bala Sai Teja Nuthalapati, Pulkit Johar, Veeresh Babu Halvi, Roopeessh Vempati

**Affiliations:** 1 Surgery, JJM Medical College, Davanagere, IND; 2 Internal Medicine, Lala Lajpat Rai Memorial Medical College, Meerut, IND; 3 Medicine, Sardar Patel Medical College, Bikaner, IND; 4 Internal Medicine, Maheshwara Medical College, Patancheru, IND; 5 Anaesthesiology, All India Institute of Medical Sciences, Rishikesh, IND; 6 Medicine, Government General Hospital, Kurnool, IND; 7 Internal Medicine, Gandhi Medical College and Hospital, Secunderabad, IND

**Keywords:** holistic patient care, emergency medicine, cardiac anomalies, neurocardiology, multidisciplinary approach, congenitally corrected transposition of great arteries (cctga), acute stroke

## Abstract

Acute stroke and the transposition of great arteries are two distinct medical entities that rarely intersect in clinical practice. Acute stroke, a devastating neurological event, occurs due to a sudden interruption of blood flow to the brain, leading to focal neurological deficits. On the other hand, the transposition of great arteries is a congenital heart defect characterized by a complete reversal of the aorta and pulmonary artery, resulting in abnormal blood circulation. Traditionally, transposition of great arteries is diagnosed in infancy and managed with surgical interventions. However, instances of this condition being discovered in adulthood are exceedingly rare.

We present the case of a 35-year-old male who presented to the emergency department with acute stroke symptoms such as sudden-onset left-sided weakness and speech difficulties. Upon further investigation, we uncovered an unexpected finding of congenitally corrected transposition of great arteries, a congenital heart defect usually diagnosed in infancy. The patient's medical history was unremarkable for cardiovascular issues, making this association even more intriguing.

The clinical course of the patient involved immediate management of the acute stroke, followed by comprehensive cardiac evaluations to assess the implications of the transposition of great arteries. Cardiac imaging revealed anatomical variations and hemodynamic consequences, prompting a multidisciplinary approach to address both conditions.

## Introduction

Amidst the intricate structure of the human heart, there exists a rare anomaly that challenges our understanding of cardiovascular complexity - congenitally corrected transposition of the great arteries (CCTGA), also known as L-transposition, is a rare and complex congenital heart defect (CHD). It has an estimated prevalence of one per 33,000 live births, accounting for approximately only 0.05% of all CHDs. CCTGA is characterized by an inversion of the ventricles and abnormal positioning of the great arteries [1]. The core anomaly involves discordant atrioventricular and ventriculo-arterial connections. Specifically, the right atrium is linked to the left ventricle via the mitral valve, while the left atrium connects to the right ventricle via the tricuspid valve. Aorta-pulmonary connections vary, with the left ventricle typically positioned on the right and the aorta arising from the right ventricle. In some cases, this arrangement is reversed. This discordance in atrioventricular and ventriculo-arterial connections leads to a fundamentally distinct circulation from normal anatomy and is also described as "double discordance" [2].

The natural course of CCTGA is crucial post-birth, especially without heart block or failure. Repairing associated malformations is possible unless severe tricuspid regurgitation (TR) determines the mortality rate [3]. Congestive heart failure (CHF) and systemic ventricular dysfunction were common in these patients, both with and without associated cardiac lesions. By age 45, 67% of patients with associated lesions had CHF, and 25% of patients without associated lesions experienced this complication. The rates of systemic ventricular dysfunction and CHF were higher with increasing age [4].

While CCTGA itself presents significant cardiovascular challenges, its association with acute cerebrovascular events, such as stroke, remains a relatively unexplored and intriguing area of study. This report highlights a unique case: a CCTGA patient with an acute stroke. We aim to understand the connection between congenital heart disease and cerebrovascular events, stressing interdisciplinary collaboration for better care and outcomes. Further research into this link is crucial.

## Case presentation

A 34-year-old gentleman presented to our emergency department with complaints of breathlessness and weakness in the left upper and lower limbs since the morning. He exhibited facial asymmetry and slurred speech, symptoms that he reported also began that morning. The patient disclosed a persistent cough and fever history over the past eight days.

Upon examination, the patient was conscious, alert, and oriented. His vital signs were recorded: blood pressure of 118/86 mmHg, pulse rate of 104 beats per minute, and oxygen saturation at 92%. Systemic examination did not reveal any notable abnormalities. Notably, the patient had a history of smoking but denied alcohol consumption, and he was not currently on any medications.

**Figure 1:**
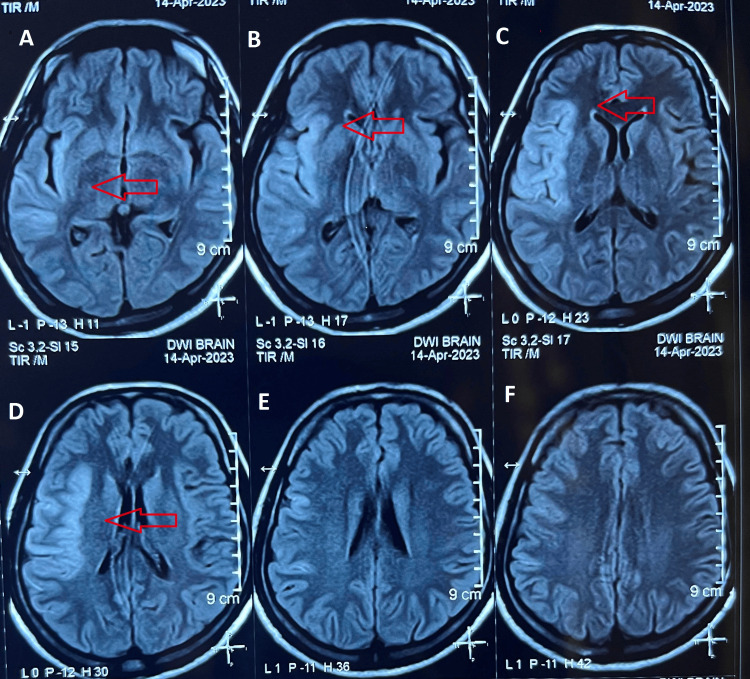
DWI MRI of Brain the showing acute infarct in the right parieto-temporal lobe and insular cortex. A, B, C, D, E, F: Showing infarction in parietal lobe DWI: Diffusion-Weighted Imaging, MRI: Magnetic Resonance Imaging

**Figure 2:**
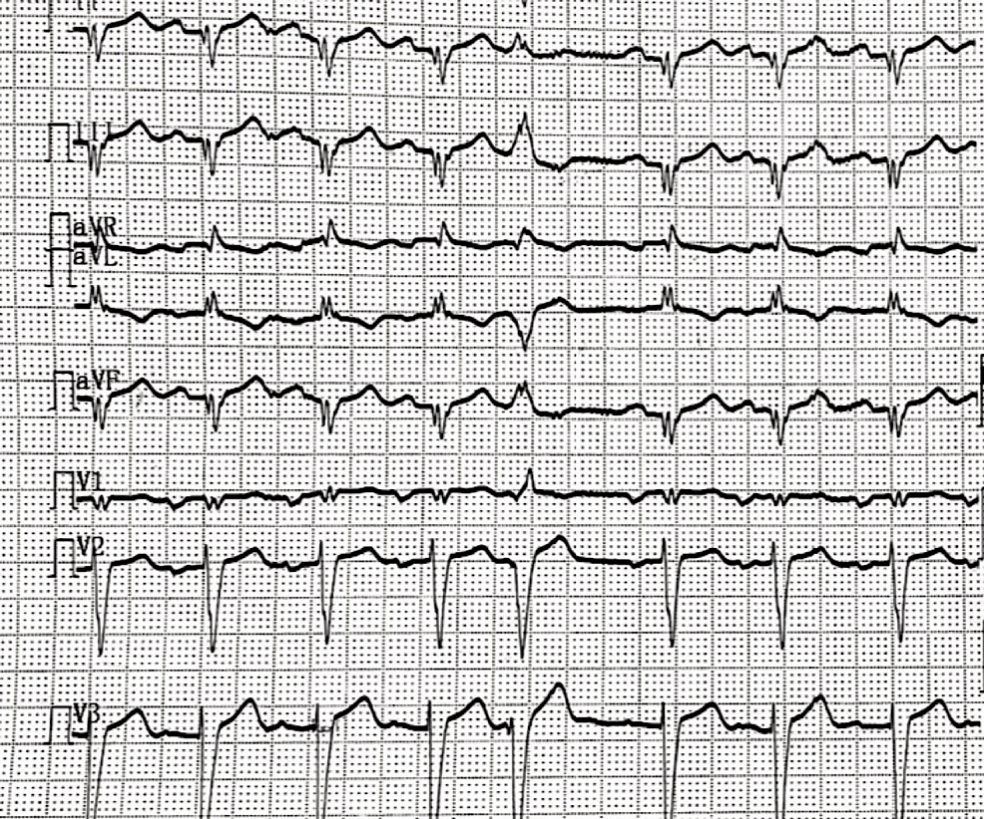
Electrocardiogram (ECG) findings exhibiting poor R-wave progression

**Figure 3:**
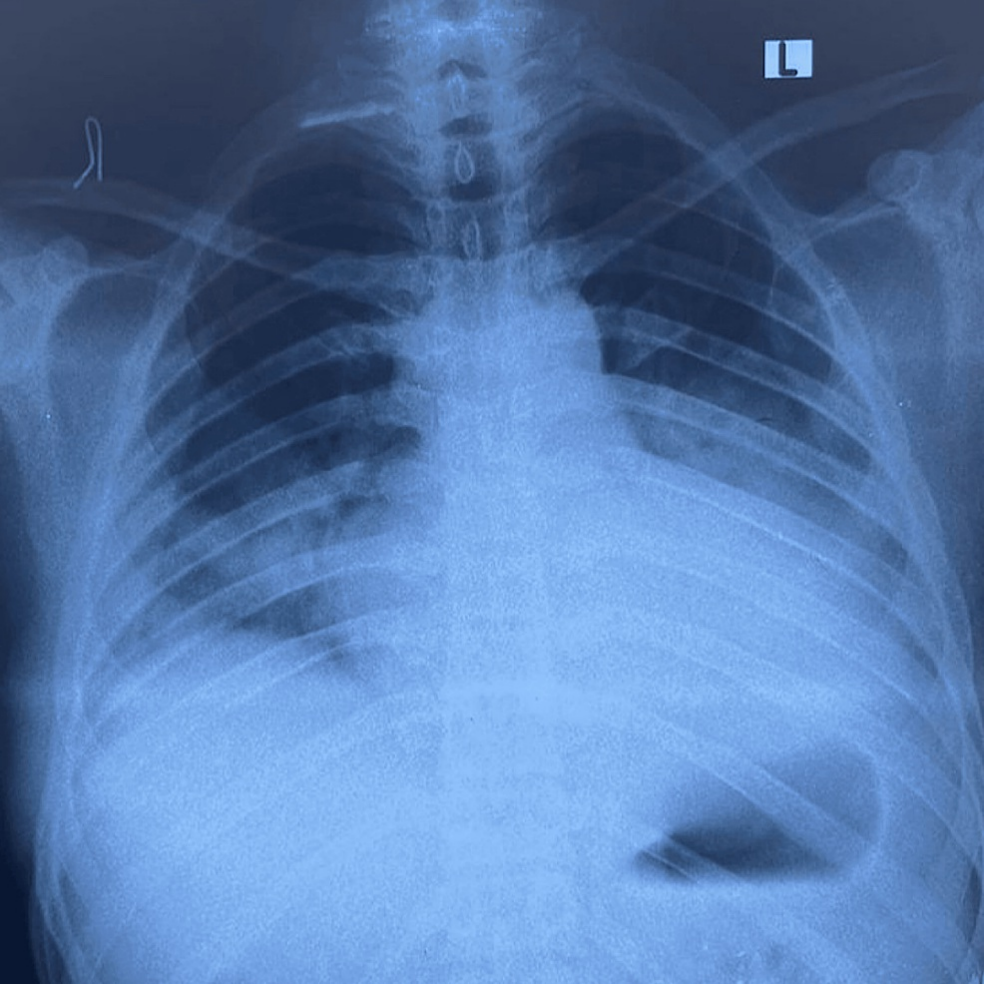
Chest imaging showing cardiomegaly

Diagnostic investigations were initiated in light of the acute neurological symptoms. Magnetic resonance imaging (MRI) revealed an acute infarct in the right parietal lobe and insular cortex, consistent with the clinical presentation (Figure 1). Electrocardiogram (ECG) findings exhibited poor R-wave progression (Figure 2). A chest X-ray demonstrated cardiomegaly, further prompting the need for cardiac evaluation (Figure 3).

**Figure 4:**
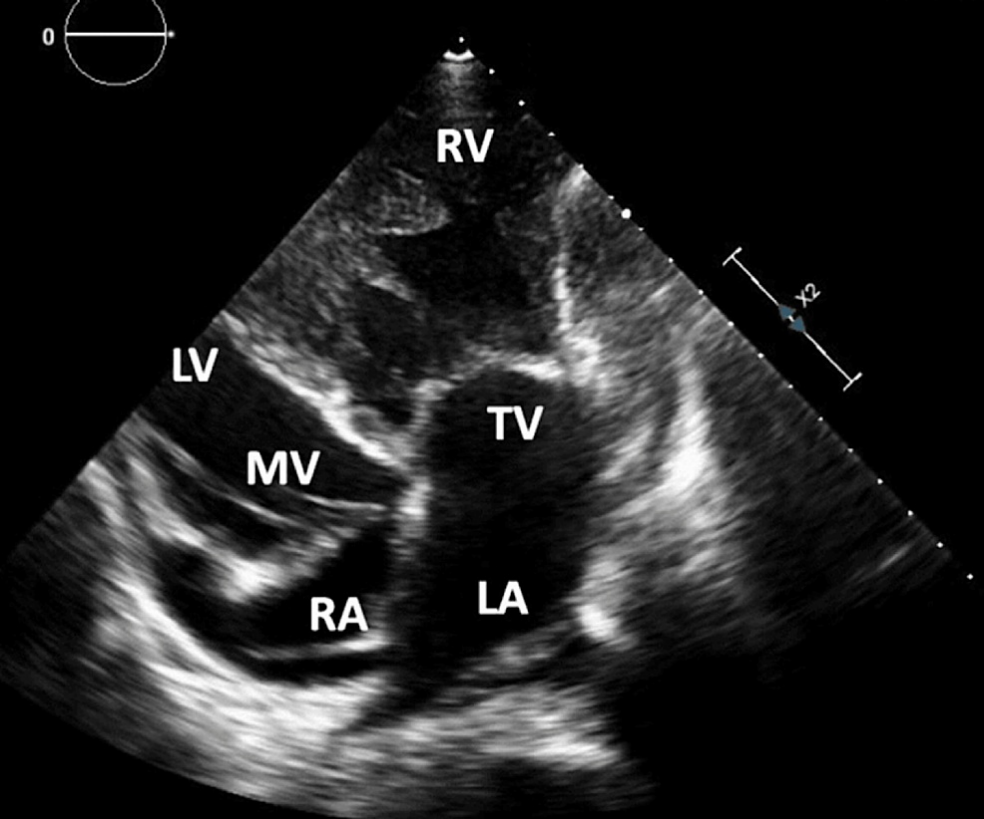
Echocardiography (ECHO) Evaluation of Cardiac Structure and Function in Congenitally Corrected Transposition of the Great Arteries (CCTGA) Congenitally corrected transposition of the great arteries featuring the tricuspid valve situated more apically on the right, which interfaces with a hypertrophied right ventricle.

Given the complexity of the presentation, including the acute neurological symptoms and the cardiac abnormalities detected on ECG and chest X-ray, a comprehensive diagnostic approach was warranted. Echocardiography (ECHO) was expeditiously conducted to assess cardiac structure and function (Figure 4). The ECHO results unveiled unexpected and significant findings. The patient exhibited dilated left-sided chambers, hypoplastic right-sided chambers, atrioventricular (AV) and ventriculoarterial (VA) discordance suggestive of CCTGA. Moreover, the ECHO disclosed moderate left atrioventricular valve regurgitation and global hypokinesis of the left ventricle. The patient's ejection fraction was measured at 30%, indicating impaired cardiac function. Mild pericardial effusion was also noted. Further contributing to the complexity of the cardiac anatomy, the aorta was dextroposed.

The management plan was comprehensively tailored to address the multifaceted clinical presentation. Fluid restriction was advised to alleviate cardiac strain, antiplatelet therapy was initiated to mitigate the risk of thrombotic events following the stroke, anticoagulants were recommended to prevent intracardiac clot formation, and diuretics were prescribed to address fluid retention and congestion. These therapeutic interventions were carefully selected to manage the acute neurological event and the underlying cardiac anomaly.

## Discussion

The most common abnormalities associated with CCTGA in middle-aged patients are systemic ventricular dysfunction and clinical congestive heart failure. Life expectancy of patients with CCTGA is reduced compared to the general population and most of the patients experience cardiac failure by the fourth or fifth decade of life. In those with ventricular enlargement and cardiac failure, vasodilator therapy is warranted [4]. Zimmerman et al. reported a case of a patient who presented with acute myocardial infarction who had a congenitally corrected transposition of the great arteries [5]. Tandon et al. presented another case of dextroversion CTGA in a patient who presented with cocaine-induced acute myocardial infarction [6]. Survival with CCTGA has been reported until the seventh and eighth decades of life. A common outcome reported is subaortic ventricular failure early in life, associated with systemic AV valve (SAVV) regurgitation. Associated abnormalities are common, but most frequent among them are pulmonary stenosis (valvular or subvalvular) (40%-74%), ventricular septal defect (70%), systemic atrioventricular (tricuspid) valve abnormalities (90%), and complete heart block (2%) [7]. Due to fibrosis of the intrinsic conduction system, the risk of developing heart block in these patients increases by 2% annually [7]. Baron Rokitansky was the first to describe CCTGA which is also known as L-transposition or ventricular inversion or double discordance. CCTGA occurs in one in 33,000 live births and has double discordance, both atrio-ventricular and ventriculo-arterial discordance [8]. Even though the right ventricle is subaortic in position, it results in a physiologically normal blood flow. CCTGA is usually associated with defects like ventricular septal defect (VSD), pulmonary stenosis (PS), etc. Only 1% of cases are not associated with any such defects [9]. Unlike many congenital heart defects that are diagnosed in infancy due to their significant impact on circulatory dynamics, CCTGA often remains undiagnosed for decades. The subtle and nonspecific symptoms, combined with the absence of commonly associated cardiac anomalies, can lead to missed diagnoses. This phenomenon prompts us to examine the current type of patients with diagnostic tests like chest X-rays, 2D echo, and MRIs, underscoring the need for heightened awareness regarding CCTGA among healthcare providers. 2D echo being a non-invasive and readily available imaging modality is usually the first investigation done. Cardiac MRI is usually the gold standard imaging modality. Cardiac catheterization may be required in older individuals with CCTGA to delineate the discordant cardiac anatomy and visualize cardiac lesions and obstructions.

Morphologic RV function is one of the key prognostic factors for patients with CCTGA. The right ventricle is traditionally not accustomed to the high pressure of the systemic circulation but in patients with CCTGA, it is exposed to it. This could be one of the plausible causes of heart failure in these patients. We believe it could also be one of the plausible causes that lead to ischemic stroke in our patient. Patients diagnosed with CCTGA can be managed conservatively or through a surgical approach. The conservative approach involves reduction of afterload and hence improvement in heart failure symptoms through vasodilators, angiotensin-converting enzyme (ACE) inhibitors, angiotensin II receptor blockers (ARBs), diuretics etc. Double switch operation characterized by a Senning/Mustard operation plus a Rastelli or Jatene operation is usually done when CCTGA is detected in children [10]. There is no robust data yet that these operations can be successfully done in adults, although they have been performed in adults, too. In this case, also the patient has been suggested a surgical procedure but due to the patient's financial status and his decision we had to manage it conservatively. Heart transplant is another option that can be considered but one needs to weigh the risks and benefits involved.

The presented case of a 35-year-old male with acute stroke symptoms uncovering CCTGA indeed raises intriguing points for discussion. Per our knowledge, this is the first case of CCTGA presenting with stroke reported in the literature. The rarity of such an occurrence highlights the complex interplay between cardiovascular and neurological systems, prompting considerations across various disciplines. The case emphasizes the importance of imaging in providing a valuable differential diagnosis and having a high degree of clinical suspicion in an unusual presentation of a condition.

The rarity of adult-onset stroke with CCTGA presents a unique opportunity for further research. Investigating the molecular, genetic, and physiological factors contributing to delayed symptom onset could provide insights into new therapeutic avenues and research opportunities. Exploring the potential benefits of early detection and intervention in asymptomatic individuals could have far-reaching implications for patient outcomes and quality of life.

## Conclusions

This case highlights the rare occurrence of a 35-year-old male presenting with acute stroke symptoms attributed to a congenital heart anomaly, specifically transposition of the great arteries. The unexpected nature of this diagnosis, coupled with the absence of prior cardiovascular history, underscores the importance of considering atypical etiologies in stroke cases. The multidisciplinary approach employed, involving both neurology and cardiology expertise, allowed for prompt management of the acute stroke and subsequent comprehensive evaluation of the congenital heart defect's anatomical and hemodynamic implications. The significance of this case lies not only in its clinical uniqueness but also in its implications for patient care. Clinicians must remain vigilant to the possibility of congenital heart defects in adult patients presenting with stroke symptoms, notably when traditional risk factors are lacking. Collaboration between specialities is essential in determining appropriate treatment strategies and long-term management plans for patients with complex comorbidities. As medical understanding evolves, this case serves as a reminder that seemingly unrelated conditions can coexist, necessitating thorough investigation and holistic patient care. Further research and case studies in similar scenarios will contribute to a deeper comprehension of the interplay between congenital heart defects and cerebrovascular events, ultimately guiding improved diagnostic and therapeutic approaches for affected individuals.
